# Huang Gan Formula Eliminates the Oxidative Stress Effects of Advanced Oxidation Protein Products on the Divergent Regulation of the Expression of AGEs Receptors via the JAK2/STAT3 Pathway

**DOI:** 10.1155/2017/4520916

**Published:** 2017-03-30

**Authors:** Quanwen Deng, Can Bu, Liqian Mo, Bin Lv, Shaolian Song, Xiaoyan Xiao, Guo Dan, Xixiao Yang

**Affiliations:** ^1^Department of Pharmacy, Nanfang Hospital, Southern Medical University, Guangzhou 510515, China; ^2^Department of Pharmacy, Shenzhen Hospital, Southern Medical University, Shenzhen 518000, China

## Abstract

Chronic kidney disease (CKD) has a high prevalence and low cure rate and represents a significant health issue. Oxidative stress is common in CKD due to metabolic disorders, inflammation, and impaired renal function changing normal proteins into advanced oxidation protein products (AOPPs). Huang Gan formula (HGF) is a new type of traditional Chinese herbal medicine. Although we previously investigated the protective effects of HGF against oxidative stress, the mechanism of HGF in CKD is still not fully understood. In this study, we used western blotting, quantitative polymerase chain reaction, and biochemical assays to show that HGF significantly decreased AOPP-induced oxidative stress damage. Moreover, the protective effects of HGF might be associated with upregulation of the advanced glycation end product receptor 1 (AGE-R1) and downregulation of the receptor for advance glycation end products (RAGE). Treatment with HGF and the Janus kinase 2 (JAK2) inhibitor, AG4-90, significantly attenuated AOPP-induced JAK2/STAT3 protein levels. These findings indicate that HGF inhibits AOPP-mediated biological responses by inactivating the JAK2/STAT3 pathway. In conclusion, HGF eliminated AOPP-induced effects in human mesangial cells (HMCs) by interrupting JAK2/STAT3 signaling, which altered RAGE/AGE-R1 expression and reduced oxidative stress in CKD.

## 1. Introduction

Chronic kidney disease (CKD) is a clinical disorder with high prevalence and low cure rate. CKD causes changes in renal structure and renal dysfunction [1]. Oxidative stress is prevalent in patients with chronic renal failure. This causes an imbalance between free radicals and nitrogen species (RNS) and reactive oxygen species (ROS) [2] and increases antioxidant-free radicals, which impair defense mechanisms [3]. ROS contribute significantly to oxidative stress in complex biological systems. Prolonged oxidative stress causes oxidization of plasma proteins into lipoperoxidation products and advanced oxidation protein products (AOPPs) [4]. High levels of lipoperoxidation products and AOPPs lead to renal insufficiency [3].

AOPPs commonly accumulate in the kidney and plasma of CKD patients [4, 5]. AOPPs are a family of oxidized protein compounds that contain dityrosine. They cross-link the protein products of oxidative stress created by the reaction of plasma protein with chlorinated oxidants. These products are pathogenic mediators of renal injury [6, 7]. Moreover, AOPPs accumulation has been reported in diabetes and CKD and has been associated with the deposition of mesangial extracellular matrix (ECM) and progressive glomerulosclerosis [4, 8]. Emerging evidence has implicated AOPPs in renal pathogenesis, but the underlying mechanisms remain unclear. The advanced glycation end product receptor (AGER) has different functions in the toxicity and disposal (detoxification) of advanced glycation end products (AGEs) [9]. AGE binding receptors can be classified into two forms: (1) responding to enhanced oxidative stress, growth, and inflammation that are finely represented by AGE (RAGE) receptors and (2) detoxification of AGE including scavenger class A type II, class B type I, and AGE 1, 2, and 3 receptors. AGE receptor 1 (AGE-R1) promotes the uptake and removal of AGEs and blocks cellular AGE-mediated ROS generation and inflammation. AGE-R1 and RAGE compete for AGEs; therefore the binding of AGEs to RAGE may increase when AGE-R1 levels decrease, thereby increasing ROS levels. In this instance, RAGE signaling is unopposed by AGE-R1 [10, 11]. Overexpression of AGE-R1 in murine mesangial cells (MCs) inhibited AGE-induced cell damage and reduced basal AGE and ROS levels. This suggested that AGE-R1 downregulates AGE-induced cellular toxicity [9, 10]. Thus, increasing AGE-R1 expression and decreasing RAGE expression may slow the progression of CKD. The JAK/STAT pathway mediates many cellular responses. Previous studies have indicated that activation of Janus kinase (JAK) signal transduction as well as transcription (STAT) pathways promotes the proliferation of some extrarenal cell lines [3, 12, 13]. AOPP accumulation in the plasma of CKD patients causes oxidative stress and affects AGER expression. However, the effects of AOPPs on AGERs remain unclear.

In recent years, traditional Chinese medicine has played an important role in the treatment of many diseases. Niaoduqing particles have been developed by the Southern Medical University Southern Hospital and have been used as the primary treatment to prevent CKD progression. Huang Gan formula (HGF) is a second generation of Niaoduqing particle products. It is a novel formula based on the theories of traditional Chinese medicine. HGF compound was chemically characterized by HPLC–Q-TOF-MS spectrometry. Eight major compounds were found and measured by comparison with reference standards [14]. We previously showed that HGF significantly decreased oxidative stress, improved renal function, and delayed renal fibrosis in rat models of CKD caused by adenine or 5/6 nephrectomy [14]. However, the precise mechanisms by which HGF regulates RAGE expression have not been fully clarified. In this study, we investigated the ability of HGF to eliminate AOPP-induced oxidative stress. In addition, we examined the underlying mechanisms, specifically whether AOPPs affect AGE receptor expression via JAK2/STAT3 signaling.

## 2. Materials and Methods

### 2.1. Reagents

BCA protein assay was obtained from Key Gen Biotech (Nanjing, China). Superoxide dismutase (SOD), malondialdehyde (MDA), and the reduced glutathione (GSH) assay were obtained from Nanjing Jiancheng Institution of Biotechnology (Nanjing, China). Cytotoxicity LDH Assay kit was obtained from WST (Dongren, Japan). 2′,7′-Dichlorofluorescin diacetate (DCFH-DA), fetal bovine serum (FBS), and Dulbecco's modified Eagle medium (DMEM) (high glucose) were purchased from Gibco (Grand Island, NY, USA). AG-490 was obtained from Calbiochem Corp (San Diego, CA, USA). The one-step RT-PCR kit (DRR036A) and SYBR Premix Taq kit (DRR820A) were purchased from Ta Ka Ra Biotech Co. (Dalian, China). Other antibodies included a rabbit polyclonal anti-RAGE antibody (BS6719; Bioworld Technology, Inc., MN, USA), anti-OST48 (H-300) antibody (sc-25558; Santa Cruz Biotechnology, Inc., CA, USA), and anti-glyceraldehyde 3-phosphate dehydrogenase (GAPDH) antibody (ab9485; Abcam). Antibodies against JAK2, STAT3, phosphotyro-JAK2, and phosphotyro-STAT3 were obtained from Affinity Bioreagents (AF6294; AF3024; AF6022; AF3295, NY, USA). All other chemicals were purchased from Sigma (USA).

### 2.2. Plant Materials and HGF Preparation

HGF consists of five herbs, including Radix et Rhizoma Rhei (307.1 g), Rhizoma Zingiberis (229.9 g), Radix Bupleuri (614.3 g), Radix Glycyrrhizae (307.1 g), and Radix Aconiti Lateralis Preparata (245.7 g). All compounds were supplied by Kangmei Pharmaceutical Co., Ltd (Guangdong, China) and were identified by Professor Hongwei Zhang of the School of Traditional Chinese Medicine at Southern Medical University. HGF was prepared according to our previous study [14].

### 2.3. Cell Culture

Human mesangial cells (HMCs) were supplied by American Type Culture Collection (Manassas, USA). HMCs were maintained in DMEM containing 5.5 mmol/L D-glucose and 10% FBS in a humidified 5% carbon dioxide incubator at 37°C. HMCs were cultured in serum-free DMEM supplemented with the JAK2 inhibitor AG-490 for 24 hr prior to timed exposure to HGF and AOPP in some experiments. AG-490 was dissolved in DMSO [12].

### 2.4. Preparation of AOPPs

AOPP-bovine serum albumin (AOPP-BSA) was prepared as previously described. In brief, 100 mg/ml of BSA (Sigma, MO, USA) was exposed to 200 mmol/l hypochlorous acid (Fluke, Switzerland) for 30 min. Then, BSA solution was dialyzed against PBS overnight to remove free hypochlorous acid. Next, AOPP was passed through a Detoxi-Gel removal column (Pierce, USA) to remove contaminating endotoxins. The level of endotoxins was determined using an amebocyte lysate assay (Sigma). The results showed <0.025 EU/ml endotoxins. The AOPP level was also measured and the result was 0.2 + 0.02 umol/g protein in native BSA and 167.4 + 9.8 umol/g protein in AOPP-BSA [6].

### 2.5. Cell Viability Assay

The cytotoxic effects of AOPPs and HGF in HMCs were assessed by the MTT assay. Briefly, HMCs were plated at a density of 1 × 10^5^ cells per well in 96-well plates. The cells were treated with indicated doses of AOPPs and HGF and incubated for 24 h at 37°C. After incubation, 20 *μ*L of 5 mg/ml MTT solution in PBS was added to each well and the cells were incubated for 4 h at 37°C and 5% CO_2_. Then, 150 *μ*L dimethyl sulfoxide (DMSO) was added to each well. The absorbance of the purple formazan solution was quantified at 490 nm after 10 min of shaking. Cell viability was measured as a ratio of the absorbance to that in control cultures [15].

### 2.6. AOPPs Cytotoxicity Assay

The cytotoxicity of AOPPs was assessed by the LDH assay. HMCs were plated at a density of 1 × 10^5^ cells per well in 96-well plates. The cells were treated with AOPPs and incubated at 37°C for 24 h. Then, 150 *μ*L LDH release reagent was added and mixed and the cells were incubated for 1 h. The absorbance of the supernatant was measured at 490 nm and cytotoxicity was calculated according to the manufacturer's instructions.

### 2.7. Measurement of ROS Generation

Cells were treated with 2′,7′-dichlorodihydrofluorescein diacetate (Sigma-Aldrich, USA) to evaluate ROS production. Cells were cultured in 6-well plates and treated with different concentrations of AOPPs and HGF. After incubation, cells were centrifuged for 5 min at 3000 ×g. Cells were washed three times in PBS (pH 7.4). Then, cells were incubated for 30 min in 10 mmol/l 2′,7′-DCFH-DA at 37°C in the dark. Intracellular ROS formation was detected by flow cytometry at excitation/emission wavelengths of 485/530 nm [16].

### 2.8. Measurement of NO Generation

NO production was indicated by nitrite content. Cells were treated with different concentrations of AOPPs and then with HGF. Culture supernatants were collected and NO release was measured using a NO assay kit (Jiancheng, Nanjing, China), according to the manufacturer's instructions. Experiments were repeated three times. Fifty milliliters of modified Griess reagent containing 2% sulphanilamide and 0.2% N-(1-naphthyl)-ethylenediamine dihydrochloride in 5% phosphoric acid was added to 100 mL of culture medium and then incubated at room temperature for 10 min. Absorbance was measured at 540 nm using a spectrophotometer. NO content was calculated according to the manufacturer's instructions. Detection by the kit was limited to 0–800 *μ*mol/L and the sample was diluted accordingly [16].

### 2.9. MDA, GSH, and SOD Assay

A colorimetric assay was employed to measure glutathione (GSH) and superoxide dismutase (SOD) activity and malondialdehyde (MDA) release. These measurements indicated the level of oxidative damage. In brief, cells were seeded at a density of 1 × 10^5^ cells per well in 6-well plates and then cultured to the desired density. Cells were pretreated with 200 *μ*g/ml AOPPs for 12 h before treatment with various concentrations of HGF (12.5, 25, 50, 100, and 200 *μ*g/ml) for 24 h. The culture supernatant was collected and treated with kit reagents according to the manufacturer's instructions. GSH absorbance was measured at the wavelength at 490 nm. SOD absorbance was measured at the wavelength at 550 nm. MDA absorbance was measured at the wavelength at 532 nm [17].

### 2.10. RNA Isolation and Quantitative Real-Time PCR (qPCR)

Total RNA was extracted from cells using trizol (Molecular Probes). RNA was reverse transcribed using Superscript III reverse transcriptase (Invitrogen). RAGE and AGE-R1 mRNA was measured by SYBR Green qPCR assay. PCR reactions included a 30 s predenaturation phase at 95°C, a 5 s period of 40 cycles of denaturation at 95°C, followed by annealing, and a 34 s extension at 60°C. Primer sequences were as follows: RAGE, forward primer 5′-AGG AGC GTG CAG AACTGA AT, reverse primer 5′-GAG TTG GTC TGA GGC CAG AA; AGE-R1, forward primer 5′-GCT CTG ATA TCG GTG ACC CT-3′, reverse primer 5′-TCG TAG TTG TGG TGG TCG AT-3′. Fold changes after normalization to GAPDH were used to express mRNA levels. The number of transcript copies of the target gene was measured in alignment with their individual threshold cycle value [18].

### 2.11. Western Blotting Analysis

For immunoblotting, cells were treated with the desired agents for the time indicated at 37°C. Treated cells were incubated overnight in serum-free medium and then rinsed using PBS on ice. Then, cells were lysed with RIPA buffer. After cell lysis, the total protein concentration was measured using a BCA assay. Equal protein amounts per sample were separated by SDS-PAGE on 10% or 8% SDS-polyacrylamide gels. Separated proteins were transferred onto nitrocellulose membranes and blocked for 1 h in 5% dry milk in 0.1% Tween 20 in Tris-buffered saline (TTBS). After blocking, membranes were probed overnight with primary antibodies (rabbit polyclonal anti-OST48, anti-RAGE, anti-JAK2, anti-STAT3, and anti-GAPDH) in 5% dry milk in TTBS at 4°C at 1 : 2000 dilution. Then, membranes were labeled with appropriate secondary antibodies for 1 h. An enhanced chemiluminescence method (Roche) was used to detect bands.

## 3. Results

### 3.1. Influence of AOPPs and HGF on HMCs Viability

**Figure 1:**
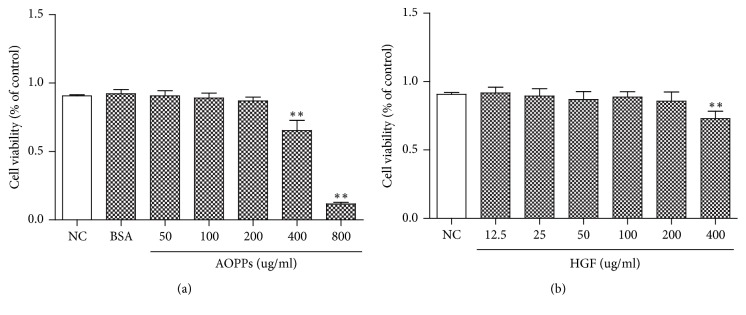
Influence of AOPPs and HGF on HMCs viability. HMCs were treated with different AOPP concentrations or 200 *μ*g/ml BSA for 24 h (a). HMCs were treated with different concentrations of HGF for 24 h (b). Data represent means ± SD; ^*∗∗*^*P* < 0.01 versus control; *n* = 3.

To determine the extent of AOPP-induced injury in HMCs, the viability of HMCs was tested using an MTT assay. AOPP concentrations lower than 200 *μ*g/ml had no significant effect on the viability of HMCs, but cell viability decreased when HGF concentrations exceeded 200 *μ*g/ml (Figure 1). Based on these findings, we selected 0–200 *μ*g/ml as the concentration range for HGF and AOPPs for further experiments.

### 3.2. Influence of AOPPs on HMCs Cytotoxicity

**Figure 2:**
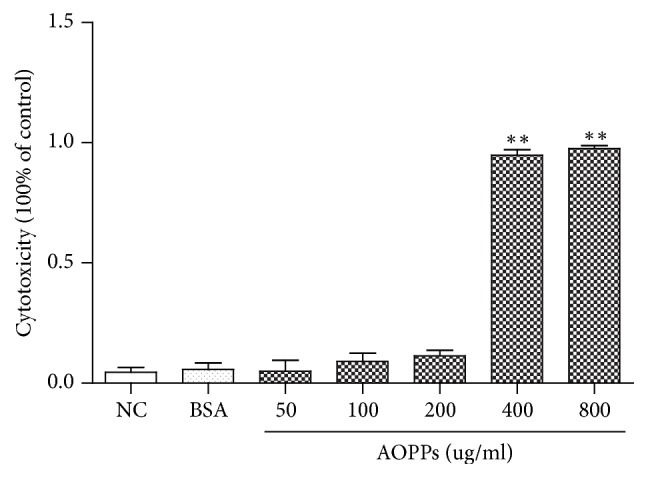
Cytotoxic effects of AOPPs on HMCs. HMCs were incubated with different concentrations of AOPPs or 200 *μ*g/ml BSA for 24 h. Data represent means ± SD; ^*∗∗*^*P* < 0.01 versus control; *n* = 3.

To determine the cytotoxic effect of AOPPs on HMCs, we performed LDH cytotoxicity assays. HMCs were treated with different AOPP concentrations for 24 h. As shown in Figure 2, AOPPs caused significant cytotoxicity in HMCs at concentrations higher than 200 *μ*g/ml. Based on these findings, 0–200 *μ*g/ml AOPP was considered a suitable concentration range for the experimental conditions.

### 3.3. Effects of AOPPs on NO and ROS Production in HMCs

**Figure 3:**
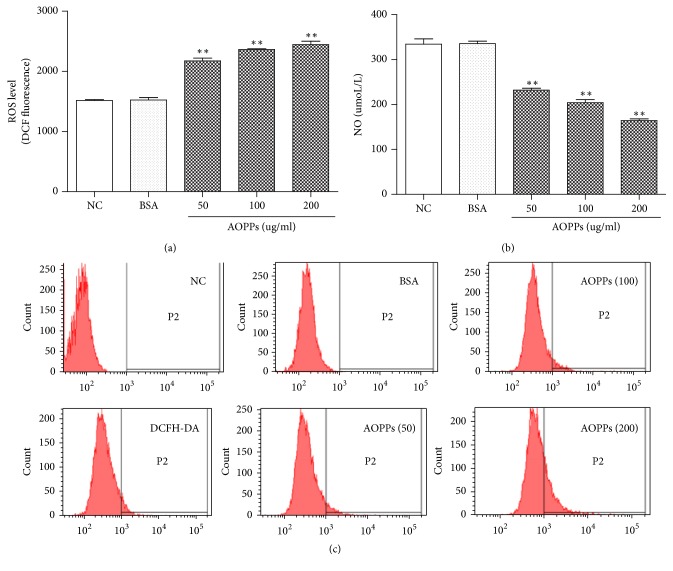
Effects of AOPPs on ROS and NO production in HMCs. The fluorescent probe DCFH-DA was used to monitor intracellular ROS. HMCs were incubated with different concentrations of AOPPs or 200 *μ*g/ml BSA for 90 min ((a) and (c)). NO production was measured by nitrite content. HMCs were treated with different concentrations of AOPPs (b). Data represent means ± SD; ^*∗∗*^*P* < 0.01 versus control; *n* = 3.

To evaluate the potential effect of AOPPs on ROS production in HMCs, we used the fluorescent probe dichlorodihydrofluorescein diacetate (DCFH-DA) to monitor ROS generation in HMCs. As shown in Figure 3(a), AOPPs (50–200 *μ*g/ml) increased ROS production in HMCs in a concentration-dependent manner 90 min after treatment. Nitrous oxide (NO) production was measured by nitrite content using NO assay kit. AOPPs (50–200 *μ*g/ml) decreased NO production in HMC cells 24 h after treatment in a concentration-dependent manner (Figure 3(b)).

### 3.4. HGF Reversed AOPP-Induced ROS and NO Production by HMCs in a Concentration-Dependent Fashion

**Figure 4:**
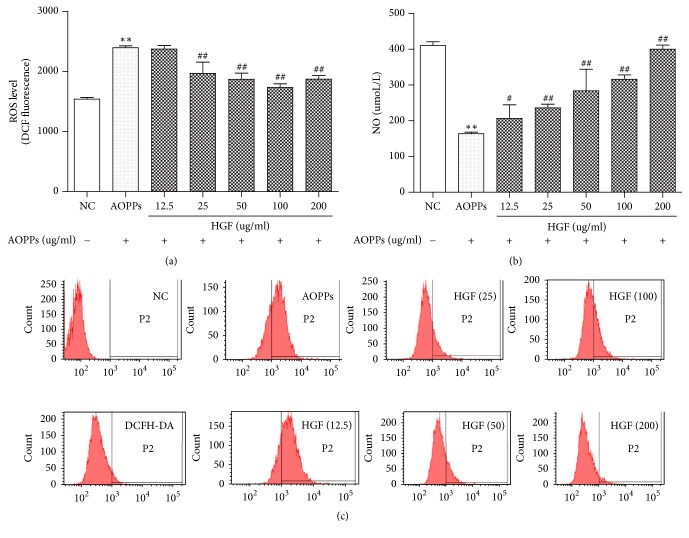
HGF reversed AOPP-induced ROS and NO production in a concentration-dependent fashion. ROS production is shown in (a) and (c) following 24 h of serum starvation. HMCs were pretreated with AOPP for 1.5 h ± HGF of the indicated concentrations for another 2 h; NO production is shown in (b). HMCs were pretreated with AOPP for 24 h ± HGF of the suggested concentrations for another 24 h. Data represent means ± SD; ^#^*P* < 0.05 versus AOPPs; ^*∗∗*^*P* < 0.01 versus control; ^##^*P* < 0.01 versus AOPPs; *n* = 3.

As shown in Figure 4(a), HGF (12.5–200 *μ*g/ml) attenuated AOPP-induced ROS production in a concentration-dependent fashion 90 min after treatment. HGF reversed AOPP-induced NO production in a concentration-dependent manner (Figure 4(b)).

### 3.5. HGF Reduces AOPPs Induced MDA Level and Increases the SOD Activity and Intracellular GSH Levels

**Figure 5:**
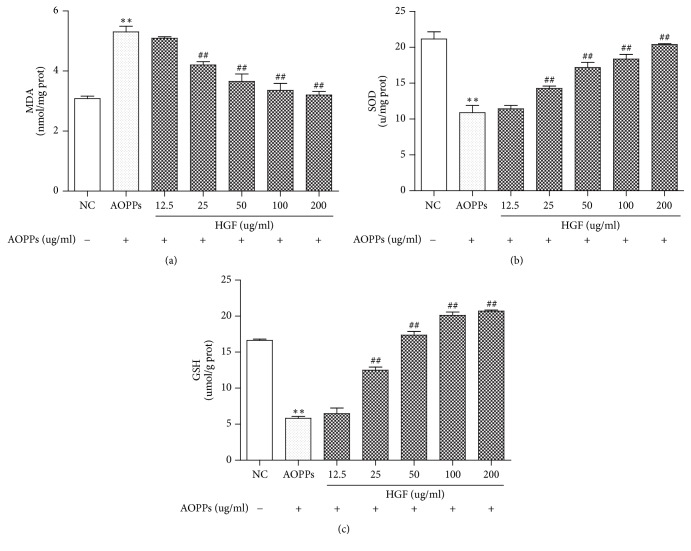
HGF reduced AOPP-induced MDA production and increased SOD activity and intracellular GSH. Cells were treated without or with AOPPs and HGF for 24 h. MDA production (a). SOD level (b). Intracellular GSH levels (c). Data represent means ± SD; ^*∗∗*^*P* < 0.01 versus control; ^##^*P* < 0.01 versus AOPPs; *n* = 3.

To determine whether HGF has a protective effect against AOPP-induced injury, we measured MDA secretion into the culture media, intracellular GSH levels, and SOD activity. MDA production was significantly elevated in the culture supernatant after 12 h exposure to AOPPs (Figure 5(a)). In contrast, SOD activity and intracellular GSH levels decreased significantly (Figures 5(b) and 5(c)). Furthermore, HGF treatment reduced MDA production and increased SOD activity and intracellular GSH compared with AOPP-treated cells in a dose-dependent manner.

### 3.6. Effects of AOPPs on JAK2 and STAT3 Phosphorylation

**Figure 6:**
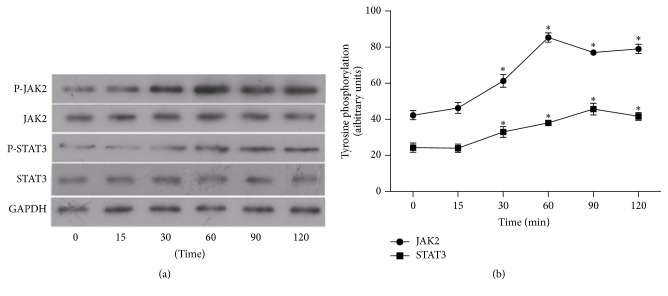
Effects of AOPPs on JAK2 and STAT3 phosphorylation. Serum-starved HMCs were incubated with 200 *μ*g/ml AOPPs for different times. Western blotting of immunoprecipitated JAK2 and STAT3 (a, b). GAPDH was used as a loading control. The data are representative of the three independent experiments. ^*∗*^*P* < 0.01 versus control.

To investigate the role of JAKs in AOPP-mediated signaling events, we measured the ability of AOPPs to phosphorylate JAK2 in HMCs. Phosphorylation of JAK kinases by AOPP (200 *μ*g/ml) was studied by immunoblotting.  Figure 6(a) shows that time-dependent phosphorylation of JAK2 was induced by AOPPs between 0 and 120 min after treatment was initiated. STATs are downstream effectors of JAK signaling; therefore we also investigated STAT3 phosphorylation in these cells. AOPPs (200 *μ*g/ml) significantly increased STAT3 phosphorylation from 0 to 2 h after treatment was initiated (Figure 6).

### 3.7. HGF Reversed AOPP-Induced RAGE and AGE-R1 Expression

**Figure 7:**
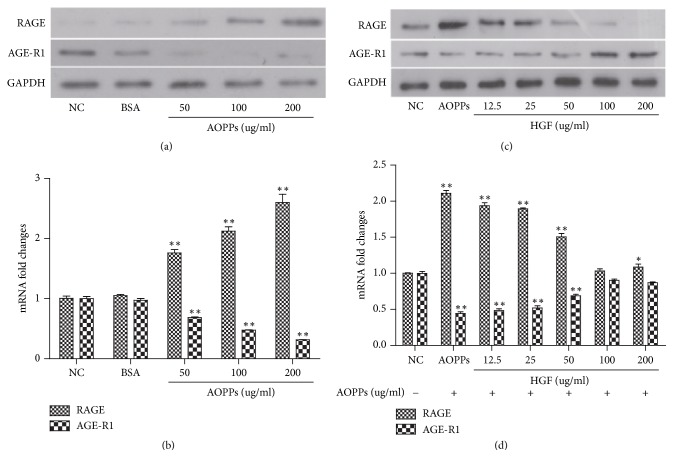
HGF reversed AOPP-mediated effects on RAGE and AGE-R1 expression. Serum-starved HMCs were treated with AOPPs at the indicated concentrations. Whole cell extracts or total RNA was prepared and RAGE and AGE-R1 expression was measured by western blotting (a) and qPCR (b). After serum starvation for 24 hr, HMCs were pretreated with AOPPs at indicated concentrations for 24 h ± with HGF for another 24 h. RAGE and AGE-R1 expression was measured by western blotting (c) and qPCR (d). GAPDH was used as a loading control. Data represent mRNA fold changes (means ± SD). ^*∗∗*^*P* < 0.01 versus untreated control (first column). Data were representatives of three independent experiments; *n* = 3.

To explore the regulatory role of AOPPs on RAGE and AGE-R1 expression, we treated HMCs with various concentrations of AOPP (0–200 *μ*g/ml). Cells were treated for 24 h under serum-starved conditions and whole cell extracts or total RNA were prepared. Compared with untreated controls (first column), AOPPs reduced AGE-R1 mRNA expression and increased RAGE expression in HMCs in a differential and dose-dependent manner (Figure 7(a)). These findings were confirmed on the protein level by western blotting (Figure 7(b)). Overall, the results suggest that AOPPs differentially regulate RAGE and AGE-R1 expression in a dose-dependent fashion in activated HMCs in vitro.

To explore the role of HGF in AOPP-mediated regulation of AGE-R1 and RAGE, serum-starved HMCs were pretreated with 200 *μ*g/ml AOPPs for 24 h before HGF treatment (12.5–200 *μ*g/ml) in serum-free media for a further 24 h. AGE-R1 and RAGE expression was quantified by western blotting (Figure 7(c)) and qPCR. HGF eliminated AOPP-mediated regulation of RAGE and AGE-R1 in HMCs (Figure 7(d)).

### 3.8. AOPPs Activated JAK2/STAT3 in HMCs, Resulting in the Divergent Regulatory Effect on RAGE and AGE-R1, Which Is Interrupted by HGF

We have demonstrated that HGF eliminates AOPP-mediated effects on AGE-R1 and RAGE expression in HMCs. However, the mechanism of this regulation was unknown. We investigated whether AOPPs regulate RAGE and AGE-R1 expression through the JAK2/STAT3 pathway and whether HGF reverses this effect through similar mechanisms. AG-490 is a tyrosine kinase inhibitor that blocks the substrate binding site of JAK2. To further examine the possibility that AOPP-dependent proliferative responses were induced by JAK2/STAT3 signaling, we treated serum-starved HMCs with (1) 200 *μ*g/ml AOPP or HGF for 24 h or (2) 10 *μ*M AG-490 for 12 h before treatment with 200 *μ*g/ml AOPPs or HGF for 24 h.

**Figure 8:**
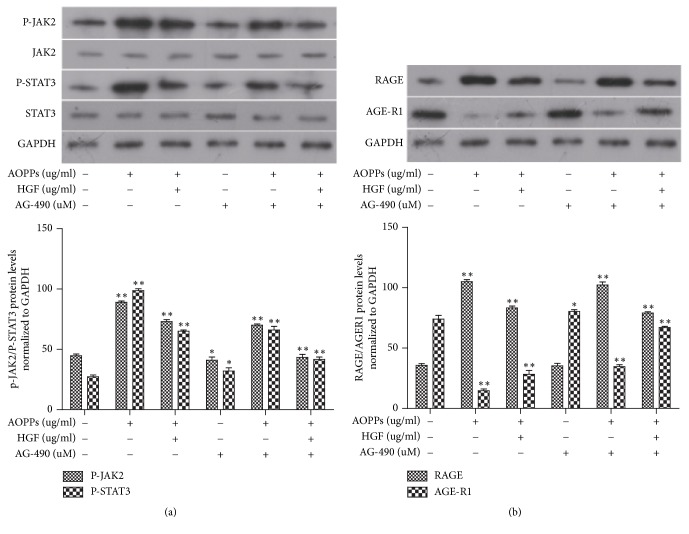
AOPPs regulate RAGE and AGE-R1 expression by activating the JAK2/STAT3 pathway in HMCs. This effect is inhibited by HGF. In one group, serum-starved HMCs were treated with AOPPs (200 *μ*g/ml) or HGF (200 *μ*g/ml) without AG-490. Cells were serum-starved for 24 h (for JAK2 detection) or 90 min (for p-JAK2 detection). Control cells were not treated. In the other group, cells were pretreated with 10 uM AG-490 for 12 h before AOPP (200 *μ*g/ml) or HGF (800 *μ*g/ml) treatment in serum-free media for a further 24 h. Western blot analysis of JAK2 and STAT3 (a). Western blot analysis of RAGE and AGE-R1 (b). GAPDH was used as a loading control. Data are representative of independent experiments. ^*∗*^*P* < 0.05 versus control (first column). ^*∗∗*^*P* < 0.01 versus control (first column).

As shown in Figure 8(a), AOPPs induced JAK2 and STAT3 phosphorylation 1.5 h after treatment compared with controls (first well or column). Inhibition of JAK2 with AG-490 and HGF treatment significantly reduced AOPP-mediated JAK2 and STAT3 phosphorylation, without affecting the expression of JAK2 and STAT3 protein.

AOPPs significantly increased RAGE expression and significantly reduced AGE-R1 expression (Figure 8(b)). These effects were apparently inhibited by HGF (corresponding to the 3rd columns and wells) and AG-490 (corresponding to the 5th columns and wells). This suggests that AOPPs regulate RAGE and AGE-R1 expression in HMCs by influencing the JAK2/STAT3 pathway. Therefore, HGF treatment may represent an effective method for reducing AOPP-mediated JAK2/STAT3 signaling.

## 4. Discussion

This research demonstrated that AOPPs caused an elevation in cellular oxidative stress and induced JAK2 and STAT3 activation in HMC, resulting in the divergent regulatory effect of RAGE and AGE-R1 expressions. Huang Gan formula did eliminate the influences of AOPPs with the interruption of JAK2/STAT3 signaling.

Oxidative stress is clinically common in patients with CKD and is responsible for CKD progression and associated complications [19]. ROS are the most significant contributing factor to oxidative stress in complex biological systems. Increased plasma AOPPs are a marker of oxidant-induced protein damage and had been reported in patients with diabetes, CKD, and chronic hepatitis. AOPPs are pathogenic mediators of many disorders. Therefore, it is important to understand how AOPPs influence cells, tissues, and organs in pathological and physiological conditions [20, 21]. Clinical trials and experimental research have suggested that AOPPs are associated with redox-dependent structural alterations associated with progressive nephropathies, including interstitial fibrosis, tubular atrophy, and glomerulosclerosis [4]. AOPPs also promote ECM overproduction and induce the fibrogenic effects of transforming growth factor-b1 [22]. In this study, we showed that AOPPs elevate MDA levels and ROS production. In contrast, AOPPs reduce SOD activity and intracellular GSH levels in HMCs. It was shown that AOPPs aggravate oxidative stress injury during CKD progression.

Plasma AOPPs concentrations were elevated in uremic patients with chronic renal failure and AOPP concentration was closely associated with AGE pentosidine levels. Both AOPP-HSA and AGE-HSA were able to trigger oxidative responses in cultured human monocytes [11]. The correlation between AGEs and AOPPs demonstrated that this relationship exists in uremic patients that have not undergone dialysis. This suggests that AGEs and AOPPs might have common biological activities and mechanisms [21, 23, 24]. The AGE receptor system, especially RAGE/AGE-R1, has been investigated as a therapeutic target for CKD. AGEs exert their influence by mediating two groups of cytoplasmic membrane receptors. RAGE enhances cell growth, oxidative stress, and inflammation [24, 25] and downregulates cellular defense mechanisms. The AGE-RAGE-oxidative stress axis is associated with the complications of cardiovascular and diabetic diseases [26]. AGE-R1 is a scavenger receptor that detoxifies and clears AGEs [27]. RAGE expression was significantly elevated in diabetic patients with high levels of AGE in the plasma [28]. In contrast, AGE-R1 was significantly reduced in diabetic patients [29]. RAGE contributes to complications associated with diabetes [18]. AGE-R1 appears to serve as a negative modulator of inflammatory responses to AGEs [27]. AOPP accumulation enhances podocyte apoptosis and depletion via RAGE [22]. Furthermore, inhibiting AGE-RAGE signaling and blocking AGE-RAGE interactions might be an effective method for treating AGE-induced complications [30]. AGE-R1 promotes AGE removal and inhibits AGE-mediated MC inflammatory injury by downregulating RAGE. This may protect injury caused by aging and diabetes in the kidney and other tissues [27]. Taken together, with the present findings, this suggests that suppressing RAGE and inducing AGE-R1 may represent novel therapeutic strategies for treating AOPP-induced CKD. JAKs and STATs transduce signals triggered by cytokines and growth factors [12]. The interaction of RAGE and AGE can stimulate JAK2/STAT3, initiating signaling cascades including leptin signaling, which control the subsequent regulation of RAGE and AGE-R1 expression [31]. We presume that AOPPs activated JAK2/STAT3 signaling in HMCs, causing the divergent regulation of RAGE and AGE-R1. We showed that HGF eliminated these AOPP-mediated effects by preventing JAK2/STAT3 activation.

According to traditional Chinese medicine, disease progression is caused by an imbalance of positive and negative regulation. At present, Chinese medical researchers believe that the AOPP-RAGE axis and RAGE signal transduction pathway play a role in CKD and that CKD progression can be prevented by blocking this signaling. However, AGE receptors receive positive and negative feedback. Therefore, regulating the balance of the AGE receptor rather than blocking ligand binding to the receptor is more in line with the theories of traditional Chinese medicine. Therefore, we propose regulating the expression of AGE receptors as a potential method of CKD treatment.

## 5. Conclusions

In summary, AOPPs increased RAGE expression, reduced AGE-R1 expression, and caused oxidative stress damage in HMCs. HGF reversed these processes. Our findings indicate that HGF may have protective effects in AOPP-mediated damage in chronic renal failure by keeping the balance of AGE receptor through regulating the JAK2/STAT3 signaling pathway. These findings introduce a new therapeutic method for treating AOPP-mediated CKD damage.

## Abbreviations

HGF: Huang Gan formula
AOPPs: Advanced oxidation protein products
RAGE: AGE receptor
AGE-R1: AGE receptor 1
AGEs: Advanced glycosylation end products
TCM: Traditional Chinese medicine
MDA: Malondialdehyde
SOD: Superoxide dismutase
GSH: L-Glutathione.
